# Effects of Tetrahydrocurcumin on Tumor Growth and Cellular Signaling in Cervical Cancer Xenografts in Nude Mice

**DOI:** 10.1155/2016/1781208

**Published:** 2016-01-04

**Authors:** Bhornprom Yoysungnoen, Parvapan Bhattarakosol, Chatchawan Changtam, Suthiluk Patumraj

**Affiliations:** ^1^Division of Physiology, Preclinical Science, Faculty of Medicine, Thammasat University, Rangsit Campus, Pathum Thani 12120, Thailand; ^2^Department of Microbiology, Faculty of Medicine, Chulalongkorn University, Bangkok 10330, Thailand; ^3^Division of Physical Science, Faculty of Science and Technology, Huachiew Chalermprakiet University, Samut Prakan 10540, Thailand; ^4^Department of Physiology, Faculty of Medicine, Chulalongkorn University, Bangkok 10330, Thailand

## Abstract

Tetrahydrocurcumin (THC) is a stable metabolite of curcumin (CUR) in physiological systems. The mechanism underlying the anticancer effect of THC is not completely understood. In the present study, we investigated the effects of THC on tumor growth and cellular signaling in cervical cancer xenografts in nude mice. Cervical cancer cells (CaSki) were subcutaneously injected in nude mice to establish tumors. One month after the injection, mice were orally administered vehicle or 100, 300, and 500 mg/kg of THC daily for 30 consecutive days. Relative tumor volume (RTV) was measured every 3-4 days. COX-2, EGFR, p-ERK1&2, p-AKT, and Ki-67 expressions were measured by immunohistochemistry whereas cell apoptosis was detected by TUNELS method. THC treatments at the doses of 100, 300, and 500 mg/kg statistically retarded the RTV by 70.40%, 76.41%, and 77.93%, respectively. The CaSki + vehicle group also showed significantly increased COX-2, EGFR, p-ERK1&2, and p-AKT; however they were attenuated by all treatments with THC. Ki-67 overexpression and a decreasing of cell apoptosis were found in CaSki + vehicle group, but these findings were reversed after the THC treatments.

## 1. Introduction

Cervical cancer is the second most common cancer in women worldwide and is the most frequent cancer in many developing countries [1]. Despite the numerous advances that have been reached in early diagnosis and treatment of cervical cancer in recent years, the prognosis of advanced/recurrent cervical cancer is rather poor. Recently, molecularly targeted therapies have dramatically improved the treatment outcomes in patients with mutant epidermal growth factor receptor (EGFR) [2]. 

EGFR is a member of the ErbB family, tyrosine kinase receptors with growth promoting effects which play a significant role in signaling pathways including cell proliferation, angiogenesis, and tumor progression [3–5]. Overexpression of EGFR signaling has been linked to the majority of cancers. Activation of EGFR has resulted in activation of MEK-extracellular signal-regulated kinase1/2 (ERK1/2) and phosphatidylinositol 3-kinase- (PI3K-) AKT pathways [6]. Several studies reported that EGFR was overexpressed in cervical biopsies of cervical cancer patients [7, 8]. Kim et al. reported that E5 oncoprotein of human papillomavirus (HPV) 16 stimulated vascular endothelial growth factor (VEGF) expression through EGFR phosphorylation [6]. Moreover, they suggested that HPV 16 E5 increases VEGF expression by activating EGFR, MEK/ERK1&2, and PI3K/AKT pathways. EGFR expression seems to have an important role in tumor angiogenesis and has been used for the detection and treatment of advanced cervical cancer.

Cyclooxygenase- (COX-) 2 is an inducible form of cyclooxygenase and is also known as prostaglandin (PG) H synthase. The expression of COX-2 is induced by various stimuli, such as growth factors and cytokines. A relationship between COX-2, its synthesized product PGE2, and cervical cancer has previously been established [9]. Sales et al. reported that COX-2, EP2, and EP4 expression and PGE2 synthesis are upregulated in cervical cancer tissue and suggest that PGE2 may regulate neoplastic cell function in cervical carcinoma in an autocrine/paracrine manner via the EP2/EP4 receptors [9]. COX-2 stimulating pathway is also dependent upon activating the MAP kinase/ERK and PI-3/AKT pathways. Agarwal et al. [10] reported that PGE_2_ upregulated the p-ERK and p-AKT levels, suggesting the involvement of ERK and AKT pathways in the 12-lipoxygenase- (LOX-) and cyclooxygenase- (COX-) 2-mediated regulation of growth in a human epidermoid carcinoma cell line (A431 cells). These findings indicated that ERK and AKT stimulating pathways were involved in the mechanism for tumor angiogenesis and tumor progression. Therefore, an appropriate target for cancer therapeutics is to explore drugs that inhibit or downregulate ERK and AKT expression.

Tetrahydrocurcumin (THC) is one of the major metabolites of curcumin (CUR) with phenolic and *β*-diketo moieties similar to CUR [11]. Previously, we demonstrated that THC showed more potent tumor antiangiogenesis activity than CUR [12], and this might be due to its possessing higher antioxidant activity. Our earlier study showed that THC demonstrated an inhibitory effect against tumor angiogenesis in CaSki-implanted nude mice, which was mediated by downregulation of HIF-1-*α*, VEGF expression, and its receptors [13]. However, the effect of THC on tumor growth or tumor progression, using cervical cancer- (CaSki-) implanted nude mice model has yet not been reported. Therefore, the present study was designed to determine the effects of THC on tumor growth in cervical cancer- (CaSki-) implanted nude mice and to study the possible mechanisms of THC on EGFR and COX-2 expression and their signaling. 

## 2. Methods

### 2.1. Cell Line and Cell Culture

Cervical cancer cells (CaSki) were purchased from the American Type Culture Collection. The cell lines were cultured in an MEM medium supplemented with 10% fetal bovine serum. All cultures were maintained in an incubator at 37°C with 5% CO_2_ in a humidified atmosphere.

### 2.2. CaSki-Induced Tumor Mice

The animal experiments were conducted according to the guidelines on experimental animals of The National Research Council of Thailand (1999). According to the procedure reported previously [13], we used BALB/c-nude female mice weighing about 20–25 g. Briefly, the mice were divided into 6 groups: (1) controls supplemented with corn oil (Control + vehicle; *n* = 6), (2) controls supplemented with THC (500 mg/kg) (Control + THC; *n* = 6), (3) CaSki-implanted mice supplemented with corn oil (CaSki + vehicle; *n* = 6), (4) CaSki-implanted mice supplemented with THC (100 mg/kg) (CaSki + THC100, *n* = 6), (5) CaSki-implanted mice supplemented with THC (300 mg/kg) (CaSki + THC300, *n* = 6), and (6) CaSki-implanted mice supplemented with THC (500 mg/kg) (CaSki + THC500, *n* = 6).

For the CaSki groups, a suspension of 10 × 10^6^ CaSki cells in 0.2 mL MEM [14] was subcutaneously injected into the dorsa of mice at the proximal midline while the Control group was injected with MEM. The tumors were measured with Vernier calipers every 3-4 days by using the formula *a* ^2^ × *b* × 0.52 (where *a* is the shortest and *b* is the longest diameter). When the tumor volume was 100–120 mm^3^, the mice were randomized. Following this, the mice were supplemented daily with vehicle or THC at the doses of 100, 300, or 500 mg/kg body weight for one month.

The tumor volume at day *n* is expressed as relative tumor volume (RTV) and calculated according to the formula RTV = TV_*n*_/TV_0_, where TV_*n*_ is the tumor volume at day *n* and TV_0_ is the tumor volume at day 0.

### 2.3. Immunohistochemistry for COX-2 and EGFR Expression

Paraffin sections from dorsal skin tissue were dewaxed and rehydrated through xylene and a graded alcohol series. Endogenous peroxidase activity was blocked with 3% hydrogen peroxide for 15 min at room temperature. After washing in water, nonspecific binding sites were blocked with 5% bovine serum in phosphate-buffered saline (PBS) for 30 min at room temperature. The tissue slide samples were incubated with primary monoclonal antibody COX-2 (Thermo Fischer Scientific, UK) (1 : 50) or primary monoclonal antibody of EGFR (VENTANA (ready to use), USA) at 4°C overnight. The slide was then gently rinsed with PBS and developed by the Envision system/HRP (DAKO Cytomation, USA) for 30 min and substrate-chromogen for 10 min at room temperature. The percent area of the COX-2 or EGFR immunoreacted to total area was analyzed by ImageJ 1.38 software (National Institutes of Health, USA).

### 2.4. Immunohistochemistry for Phospho-AKT Expression (p-AKT) and Phospho-ERK1&2 Expression (p-ERK1&2)

The tissue samples were incubated with 1 : 100 phospho-AKT (Ser473) rabbit monoclonal antibody (Cell Signaling Tech, MA, USA), or a 1 : 100 dilution phospho-p44/42 MAPK (p-ERK1&2, Thr202/Tyr204) rabbit monoclonal antibody (Cell Signaling Tech, MA, USA) overnight at 4–8°C. After washing, the sections were incubated with the Envision system/HRP (DAKO Cytomation, USA) for 30 min at room temperature. The percentage area of p-ERK1&2 and p-AKT immunoreactivated proportionate to the total area was analyzed by ImageJ 1.38 software (National Institutes of Health, USA).

### 2.5. Immunohistochemistry for Ki-67 Expression

The tissue samples were incubated with primary monoclonal antibody Ki-67 (DAKO Cytomation, USA) (Ready to use) at 4°C overnight. The slide was then gently rinsed with PBS and developed by the Envision system/HRP (DAKO Cytomation, USA) for 30 min and substrate-chromogen for 10 min at room temperature. The nuclei were counterstained with Mayer's hematoxylin. The percentage area of Ki-67 immunoreactivated proportionate to the total area was analyzed by ImageJ 1.38 software (National Institutes of Health, USA).

### 2.6. Apoptosis Analysis

Apoptotic cells in tissue samples were detected by using the ApopTag Plus Peroxidase In Situ Apoptosis Detection Kit (Millipore, USA). The deparaffinized tissue sections were permeabilized by xylene and inactivated by endogenous peroxidases with 3% H_2_O_2_ in distilled water at room temperature for 5 min. The tissue sections were incubated with TUNEL reaction mixture containing TdT at 37°C for 60 min. Slides were rinsed twice in PBS for 10 min and dried around the sample. The labeled DNA was detected by peroxidase substrate for 3–6 min at room temperature. The nuclei were counterstained immediately with Mayer's hematoxylin. Finally, the slides were washed and analyzed under light microscope. Apoptotic index (AI) was determined as the percentage of the labeled nuclei with respect to the total number of nuclei counted.

### 2.7. Statistical Analysis

Data were expressed as a mean with standard error. SPSS.13 software was used for statistical analysis. Student's unpaired *t*-test was applied for comparison of the means of two groups (Control + vehicle and Control + THC or Control + vehicle and CaSki + vehicle groups), and analysis of variance was used for the means of multiple groups. For all of the value differences, *P* value less than 0.05 was considered significant.

## 3. Results

### 3.1. Antitumor Effect of THC in CaSki-Implanted Mice

**Figure 1:**
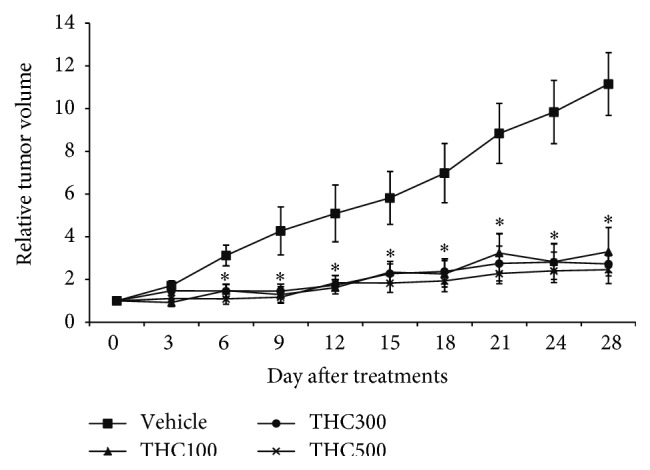
Relative tumor volume. ^*∗*^ *P* < 0.005 versus CaSki + vehicle group.

Cervical cancer (CaSki) cells were implanted in mice as described. When the tumors had attained a volume of 100–120 mm^3^, therapy was started.  Table 1 and Figure 1 represent relative tumor volume. On day 6, all treated groups showed significantly decreased relative tumor volume as compared to CaSki + vehicle group (*P* < 0.005). At the end of the experiment, THC treatments at the doses of 100, 300, and 500 mg/kg dramatically retarded the growth of tumors by 70.40%, 76.41%, and 77.93%, respectively (*P* < 0.005).

### 3.2. Effects of THC on COX-2 Expression

**Figure 2:**
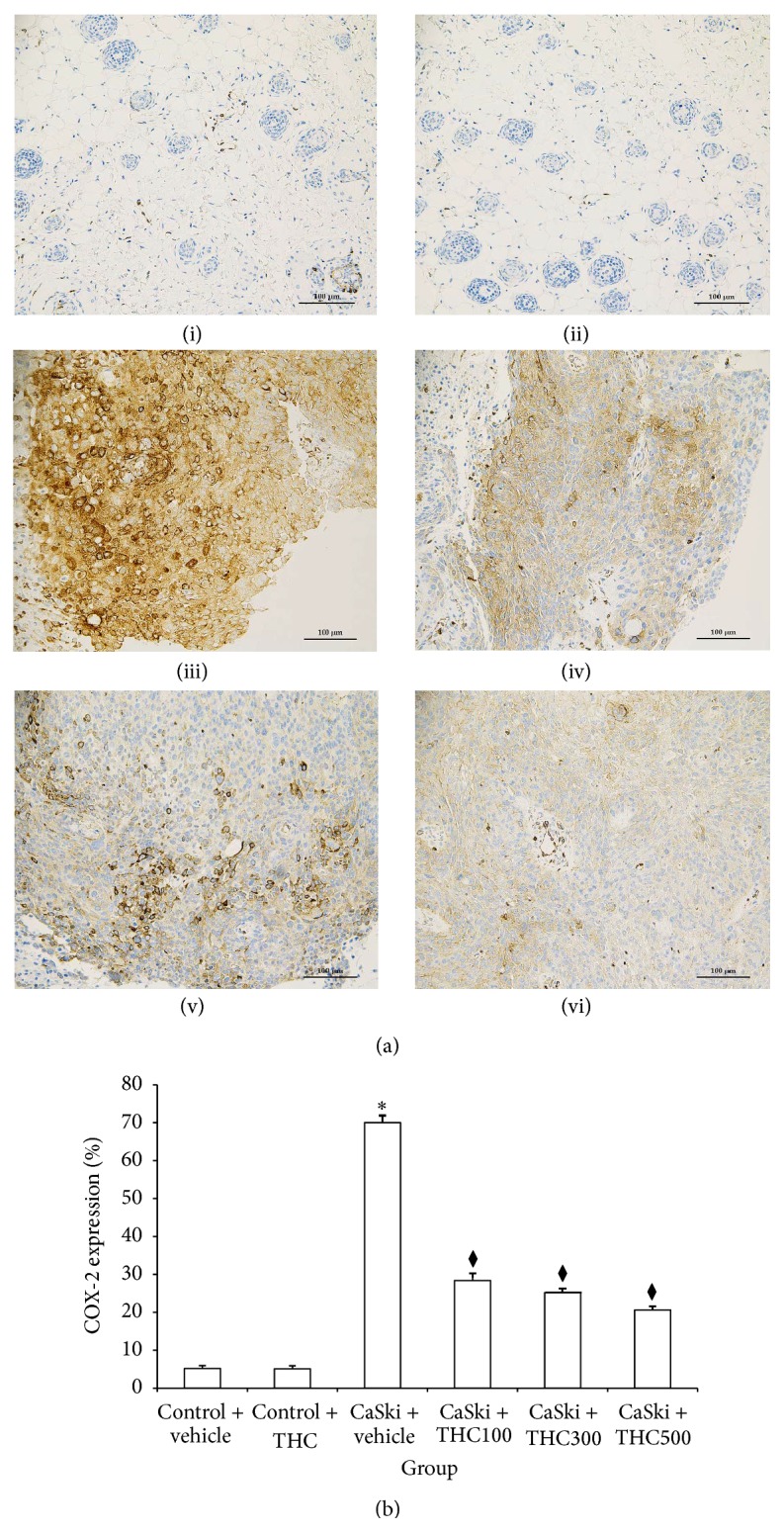
(a) COX-2 expression in Control + vehicle group (i), Control + THC group (ii), CaSki + vehicle group (iii), CaSki + THC100 group (iv), CaSki + THC300 group (v), and CaSKi + THC500 group (vi), Bar = 100 *μ*m, 200x. (b) COX-2 expression (%) (mean ± SEM). ^*∗*^ *P* < 0.001 versus Control + vehicle group; ^⧫^ *P* < 0.001 versus CaSki + vehicle group.

Figure 2(a) shows microscopic images of immunohistochemical stained sections for COX-2 expression. Stronger COX-2 expression was found in the CaSki + vehicle group than in the Control group. THC treatments attenuated COX-2 expression.

Figure 2(b) shows the expression ratio of COX-2. The expression ratio of COX-2 was significantly increased in the CaSki + vehicle group (70.00 ± 2.56%) as compared to the Control + vehicle group (5.20 ± 0.50%) (*P* < 0.001). Interestingly, the expression ratio of COX-2 was significantly reduced in all doses treated with THC (100, 300, and 500 mg/kg) (28.40 ± 1.18%; 25.20 ± 1.13%; 20.62 ± 1.08%) (*P* < 0.001).

### 3.3. Effects of THC on EGFR Expression

**Figure 3:**
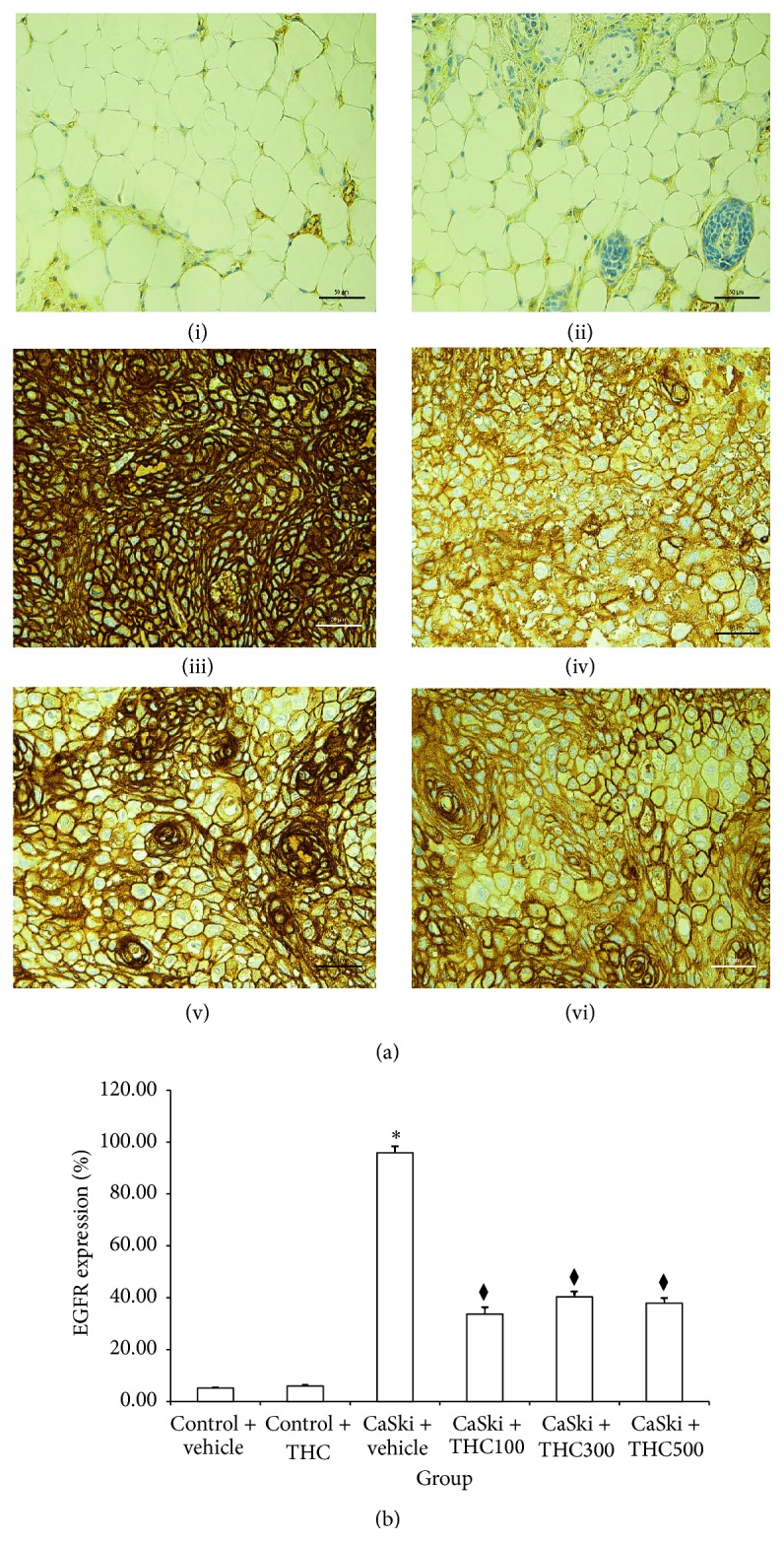
(a) EGFR expression in Control + vehicle group (i), Control + THC group (ii), CaSki + vehicle group (iii), CaSki + THC100 group (iv), CaSki + THC300 group (v), and CaSKi + THC500 group (vi), Bar = 50 *μ*m, 400x. (b) EGFR expression (%) (mean ± SEM). ^*∗*^ *P* < 0.001 versus Control + vehicle group; ^⧫^ *P* < 0.001 versus CaSki + vehicle group.

Figure 3(a) shows microscopic images of immunohistochemical stained sections for EGFR expression. The EGFR staining pattern was predominantly membrane with occasional cytoplasmic positivity. EGFR was overexpressed in CaSki + vehicle group; however it was attenuated after treatment with all doses of THC.

Figure 3(b) shows expression ratio of EGFR. The EGFR expression significantly increased in the CaSki + vehicle group (95.84 ± 2.55%) as compared to the Control + vehicle group (5.19 ± 0.32%) (*P* < 0.001), but EFGR expression decreased after treatment with all doses of THC (100, 300, and 500 mg/kg) (33.68 ± 2.76%; 40.34 ± 2.06%; 37.86 ± 1.98%) (*P* < 0.001).

### 3.4. Effects of THC on p-AKT and p-ERK1&2

**Figure 4:**
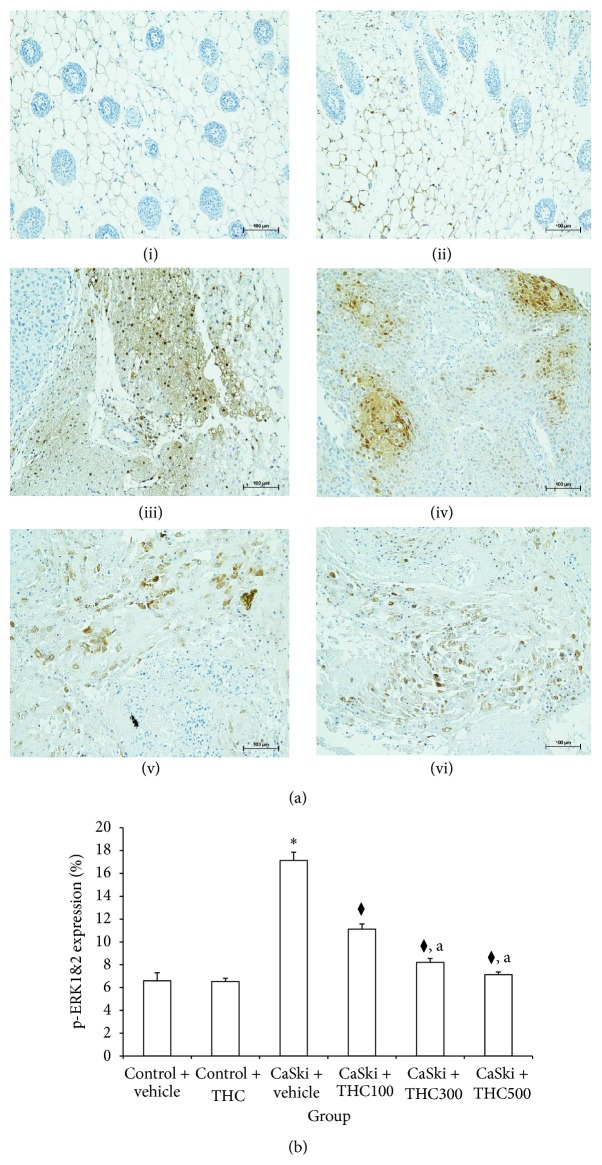
(a) p-ERK1&2 expression in Control + vehicle group (i), Control + THC group (ii), CaSki + vehicle group (iii), CaSki + THC100 group (iv), CaSki + THC300 group (v), and CaSKi + THC500 group (vi), Bar = 100 *μ*m, 200x. (b) p-ERK1&2 expression (%) (mean ± SEM). ^*∗*^ *P* < 0.001 versus Control + vehicle group, ^⧫^ *P* < 0.001 versus CaSki + vehicle group; ^a^ *P* < 0.001 versus CaSki + THC100 group.

**Figure 5:**
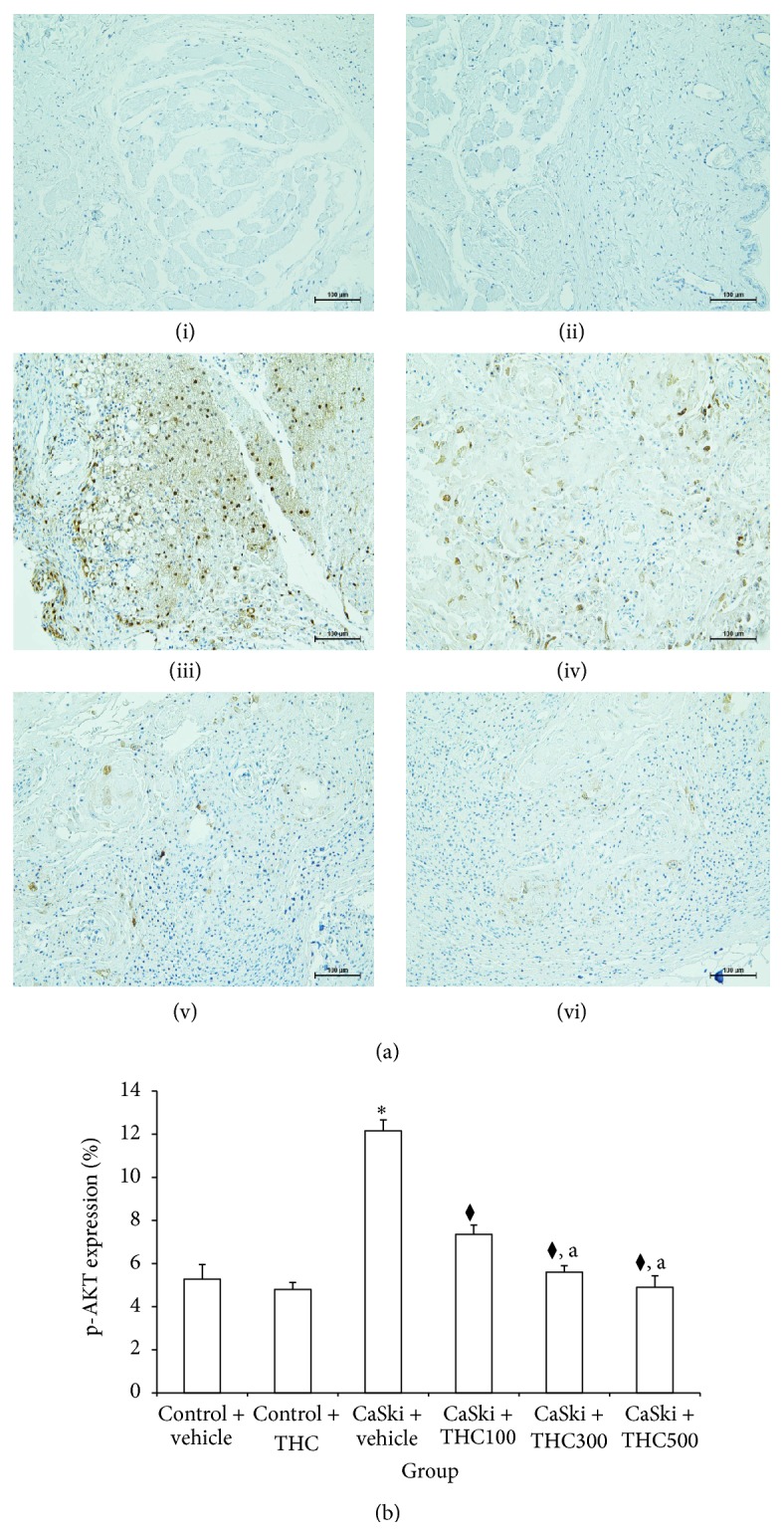
(a) p-AKT expression in Control + vehicle group (i), Control + THC group (ii), CaSki + vehicle group (iii), CaSki + THC100 group (iv), CaSki + THC300 group (v), and CaSKi + THC500 group (vi), Bar = 100 *μ*m, 200x. (b) p-AKT expression (%) (mean ± SEM). ^*∗*^ *P* < 0.001 versus Control + vehicle group, ^⧫^ *P* < 0.001 versus CaSki + vehicle group, and ^a^ *P* < 0.001 versus CaSki + THC100 group.

Figures 4(a) and 5(a) show microscopic images of immunohistochemical stained sections for p-ERK1&2 and p-AKT expression. The p-ERK1&2 and p-AKT expression revealed specific positive nuclei immunostaining. The CaSki + vehicle group exhibited overexpression of p-ERK1&2 and p-AKT, while normal tissue exhibited very low staining. All dose treatments of THC reduced the overexpression of p-ERK1&2 and p-AKT.

Figures 4(b) and 5(b) show the percent expression ratio of p-ERK1&2 and AKT. The percent expression ratio of p-ERK1&2 and AKT significantly increased in CaSki + vehicle group (17.14 ± 0.72 and 12.16 ± 0.50) as compared to the Control groups (6.59 ± 0.70 and 5.28 ± 0.68). Interestingly, THC treatment shows a significantly attenuated percent expression of p-ERK1&2 (11.12 ± 0.45%; 8.21 ± 0.34%; 7.13 ± 0.24%) (*P* < 0.001) and p-AKT (7.36 ± 0.43%; 5.60 ± 0.30%; 4.70 ± 0.53%) (*P* < 0.001). Moreover, treatments with THC at doses of 300 and 500 mg/kg showed a significantly reduced percent expression ratio of p-ERK1&2 and AKT as compared to treatment with 100 mg/kg of THC.

### 3.5. Effects of THC on Cervical Cancer Cell Proliferation (Ki67 Expression)

**Figure 6:**
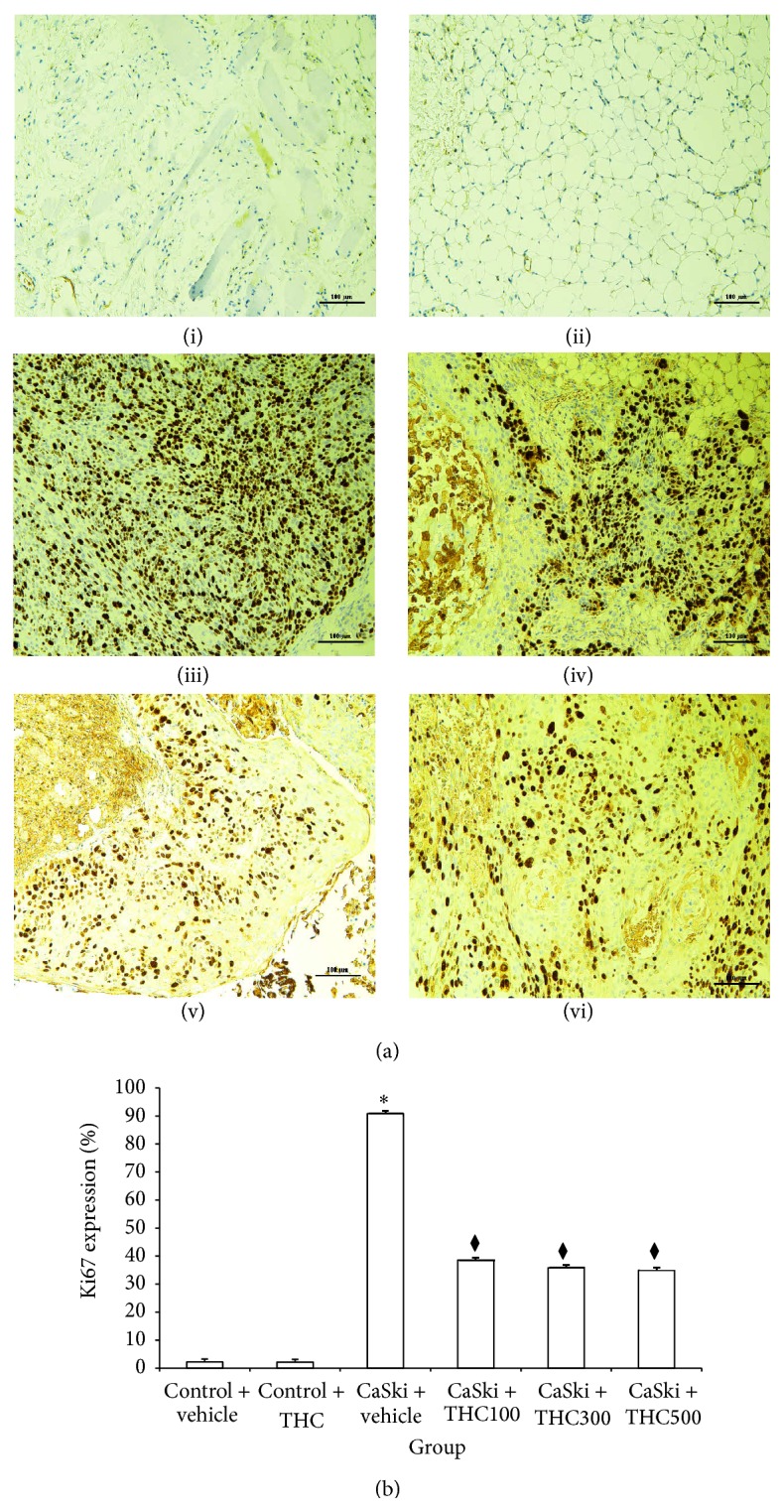
(a) Ki67 expression in Control + vehicle group (i), Control + THC group (ii), CaSki + vehicle group (iii), CaSki + THC100 group (iv), CaSki + THC300 group (v), and CaSKi + THC500 group (vi), Bar = 100 *μ*m, 200x. (b) Ki-67 expression (%) (mean ± SEM). ^*∗*^ *P* < 0.001 versus Control + vehicle group; ^⧫^ *P* < 0.001 versus CaSki + vehicle group.

Stronger Ki67 expression was found in the CaSki + vehicle group than in the Control groups (Figure 6(a)). Interestingly, our study demonstrated that all doses of THC attenuated Ki67 expression.  Figure 6(b) shows the expression ratios of Ki67. The percent of Ki67 expression significantly increased in the CaSki + vehicle group (90.86 ± 5.87%) as compared to the Control + vehicle group (2.24 ± 0.12%) (*P* < 0.001), but they were attenuated by all dose treatments with THC (100, 300, and 500 mg/kg) (38.52 ± 2.12%; 35.78 ± 1.82%; 34.92 ± 1.76%) (*P* < 0.001).

### 3.6. Effects of THC on Apoptotic Cell

**Figure 7:**
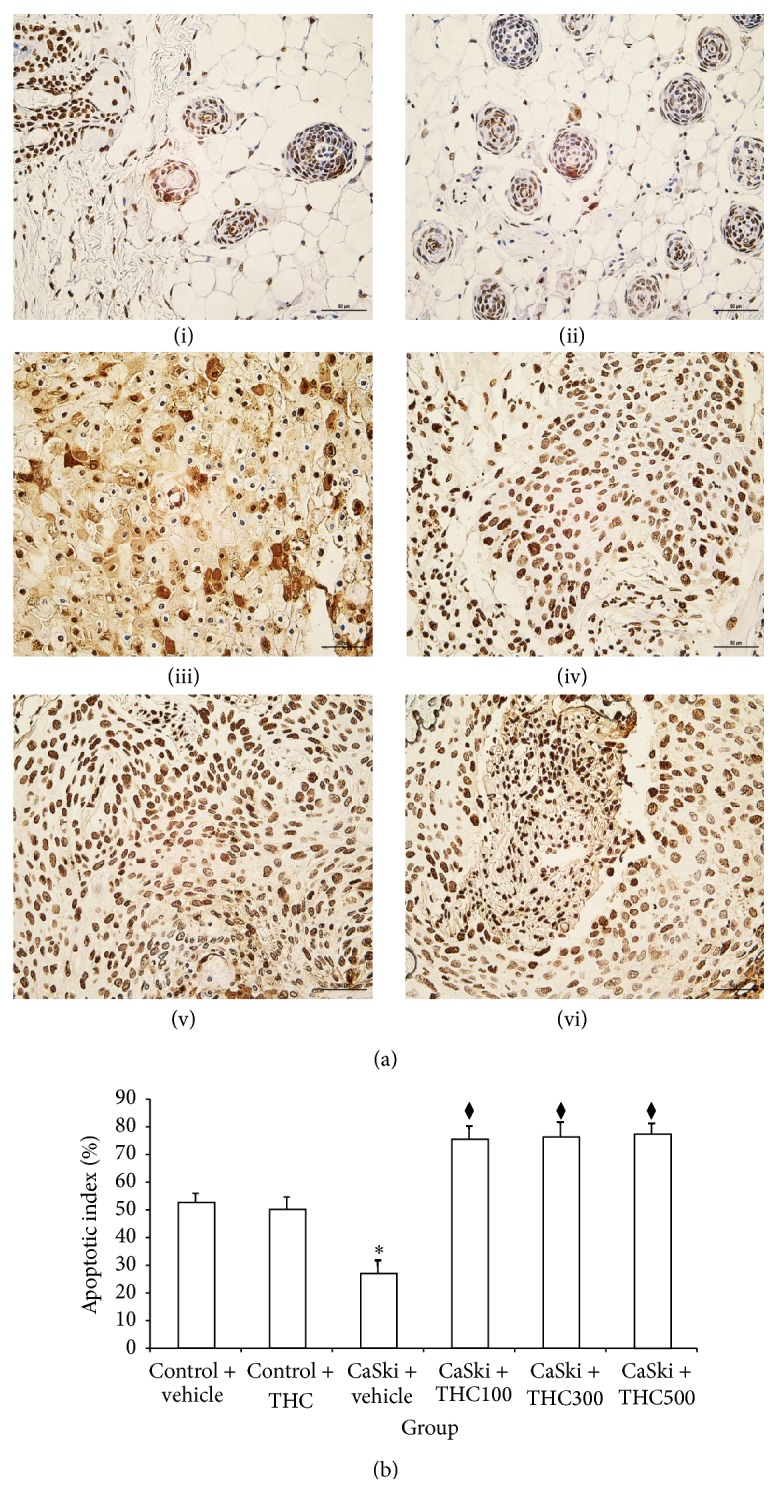
(a) Apoptotic cells in Control + vehicle group (i), Control + THC group (ii), CaSki + vehicle group (iii), CaSki + THC100 group (iv), CaSki + THC300 group (v), and CaSKi + THC500 group (vi), Bar = 50 *μ*m, 400x. (b) Apoptotic index (%) (mean ± SEM). ^*∗*^ *P* < 0.005 versus Control + vehicle group; ^⧫^ *P* < 0.005 versus CaSki + vehicle group.

Apoptotic positive cells showed up as a brown stain in almost all cells of normal dorsal skin tissue (Figure 7(a)). In contrast, the number of apoptotic positive cells was lower in the CaSki + vehicle group. Treatment with THC induced an increment of apoptotic positive cells in CaSki-induced tumor tissue. The apoptotic index (AI) was highest in the Control groups (52.75 ± 3.25 and 50.23 ± 4.42%) but was low in the CaSki + vehicle group (27.01 ± 4.75%) (Figure 7(b)). All dose treatments with THC significantly increased AI when compared to the CaSki-vehicle group (75.68 ± 4.51%; 76.45 ± 5.23%; 77.43 ± 3.84%) (*P* < 0.005). 

## 4. Discussion

The main objectives of the present study were to determine the effects of THC on tumor growth in cervical cancer- (CaSki-) implanted nude mice and to study the possible mechanisms of THC on EGFR and COX-2 expression and their signaling. We evaluated the effects of THC on tumor growth using a CaSki-implanted nude mice model. The result showed relative tumor volume dramatically increased in the CaSki + vehicle group; however, the tumor growth rate was retarded by all doses treated with THC (Table 1 and Figure 1). This has the tendency to suggest that THC treatments potentially exhibited an antitumor effect in an* in vivo* model. This study attempts to determine the mechanisms by which THC inhibits tumor growth.

Expression of COX-2 and EGFR has been demonstrated to have an important role in tumor angiogenesis in many cancers [15–20]. The association between COX-2 expression and the activation of EGFR has been shown in previous studies [20]. The enhancement of COX-2 expression is mediated through the activation of the MEK-ERK1/2 and PI3K/Akt, downstream of EGFR [20], resulting in the inhibition of apoptosis, suppression of immune function, promotion of angiogenesis, and enhancement of the invasiveness of malignant cells [21]. In the present study, CaSki cell-implanted mice were shown to display high levels of COX-2. However, the expression of COX-2 subsequently decreased after treatment with THC (Figure 2). Moreover, our data appeared to show that THC was found to attenuate the expression Ki-67, a cellular marker for cell proliferation, and induce cell apoptosis (Figures 6 and 7). Therefore, it seems that THC may inhibit tumor growth by reducing cell proliferation and inducing cell apoptosis. It has been suggested that COX-2 is an important target for chemotherapeutic effects. Our observations prompt us to suggest that inhibition of COX-2 production by THC may suppress growth and invasiveness of cervical carcinogenesis.

Overexpression of EGFR signaling has also been linked to the majority of cancers. Activation of EGFR regulates gene transcription and modulates cell proliferation, apoptosis, angiogenesis, tumor invasion, and metastasis through MEK-extracellular signal-regulated kinase1/2 (ERK1&2) kinase pathway or PI3K-AKT pathway [22]. Recently reports showed that EGFR was overexpressed in cervical biopsies of cervical cancer patients [5, 23]. The number of biopsies with intense immunoexpression of EGFR increased with the severity of cytological abnormality. In patients with squamous cell carcinoma of the cervix, this receptor is overexpressed in up to 85% of cases, and its expression has been associated with more advanced cancer stages and poor prognoses [12, 24, 25]. Sugiyama et al. [26] found that epidermal growth factor (EGF) and transforming growth factor-*α* (TGF-*α*) activated EGFR, markedly increasing COX-2 expression in a cervical carcinoma cell line. EGFR has thus been identified as a promising target for cervical cancer. In the present study, we found that EGFR was overexpressed in the CaSki + vehicle group. However, THC treatments attenuated EGFR expression (Figure 3). Moreover, p-ERK1&2 and p-AKT, the major types of signaling molecules in EGFR signaling pathway, were markedly increased in the CaSki + vehicle group as compared to the Control group. Interestingly, THC treatment could downregulate the expression of p-ERK1/2 and p-AKT (Figures 4 and 5). Since MAP kinase/ERK and PI-3/AKT pathways influence COX-2 stimulating pathway, they are likely to be involved in the mechanism for THC to inhibit tumor growth and tumor progression [10].

Currently, there is limited knowledge regarding the specific mechanism of THC against cancers. Kang et al. recently examined the efficacy and associated mechanism of the action of THC in human breast cancer, MCF-7 cells, for the first time [27]. They showed that THC inhibited cell growth by inducing MCF-7 cells to undergo mitochondrial apoptosis and G2/M arrest. The present study appears to show that THC, a reduced analog of CUR, could inhibit tumor growth and tumor progression, most likely by downregulating the expression of COX-2, EGFR, and their signaling molecules (p-ERK1&2 and p-AKT), resulting in the reduction of cell proliferation as well as promoting cellular apoptosis.

## 5. Conclusion

The current study sheds new light on our understanding of the mechanisms of THC in cervical cancer intervention. We demonstrated that THC, one of the active anticancer forms of CUR* in vivo*, markedly inhibited tumor growth and tumor progression in CaSki-implanted female nude mice models by reducing cell proliferation and promoting cell apoptosis, which is likely to have occurred through the inhibition of COX-2, EGFR, and their signaling molecules (p-ERK1&2 and p-AKT) expression. THC should be further investigated for its potential as a candidate for the development of an additional and/or alternative agent for the treatment of human cervical cancer.

## Figures and Tables

**Table 1:** Relative tumor volume (mean ± SEM). ^*∗*^ *P* < 0.005 versus CaSki + vehicle group.

Group	Day after treatments									
	0	3	6	9	12	15	18	21	24	28
CaSki + Vehicle	1	1.71 ± 0.23	3.13 ± 0.48	4.27 ± 1.12	5.09 ± 1.33	5.82 ± 1.23	6.98 ± 1.38	8.84 ± 1.40	9.84 ± 1.48	11.15 ± 1.47
CaSki + THC100	1	0.92 ± 0.19	1.48 ± 0.30^*∗*^	1.29 ± 0.37^*∗*^	1.62 ± 0.29^*∗*^	2.34 ± 0.51^*∗*^	2.26 ± 0.62^*∗*^	2.75 ± 0.90^*∗*^	2.81 ± 0.83^*∗*^	3.30 ± 1.13^*∗*^
CaSki + THC300	1	1.47 ± 0.41	1.46 ± 0.32^*∗*^	1.46 ± 0.32^*∗*^	1.74 ± 0.27^*∗*^	2.28 ± 0.45^*∗*^	2.38 ± 0.58^*∗*^	3.24 ± 0.81^*∗*^	2.83 ± 0.47^*∗*^	2.63 ± 0.39^*∗*^
CaSki + THC500	1	1.11 ± 0.16	1.09 ± 0.25^*∗*^	1.17 ± 0.27^*∗*^	1.85 ± 0.32^*∗*^	1.83 ± 0.43^*∗*^	1.93 ± 0.50^*∗*^	2.28 ± 0.47^*∗*^	2.40 ± 0.54^*∗*^	2.46 ± 0.65^*∗*^
